# Metabolomics analysis of buck semen cryopreserved with trehalose

**DOI:** 10.3389/fgene.2022.938622

**Published:** 2022-08-04

**Authors:** Bingbing Xu, Zhiying Wang, Ruijun Wang, Guoxin Song, Yanjun Zhang, Rui Su, Yongbin Liu, Jinquan Li, Jiaxin Zhang

**Affiliations:** ^1^ College of Animal Science, Inner Mongolia Agricultural University, Hohhot, China; ^2^ Inner Mongolia Key Laboratory of Animal Genetics, Breeding, and Reproduction, Hohhot, China; ^3^ Key Laboratory of Mutton Sheep Genetics and Breeding, Ministry of Agriculture and Rural Affairs, Hohhot, China; ^4^ Research Center for Animal Genetic Resources of Mongolian Plateau, College of Life Sciences, Inner Mongolia University, Hohhot, China

**Keywords:** buck, spermatozoa, cryopreservation, trehalose, metabolomics

## Abstract

Trehalose is commonly used as an impermeable cryoprotectant for cryopreservation of cells, but its cryoprotective mechanism has now not but been determined. This study investigated the cryopreservation impact of trehalose on buck semen cryopreservation and finished metabolic profiling of freeze-thawed media by way of the GC–MS-based metabolomics for the first time. Metabolic pattern recognition and metabolite identification by means of principal component analysis (PCA), partial least squares discriminant analysis (PLS-DA) and metabolic pathway topology analysis revealed the results of trehalose on buck sperm metabolism at some point of cryopreservation. The results confirmed that trehalose drastically progressed sperm motility parameters and structural integrity after thawing. PCA and PLS-DA analysis discovered that the metabolic patterns of the freezing-thawing media of buck semen cryopreserved with trehalose (T group) or without trehalose (G group, Control) were certainly separated. Using screening conditions of VIP >1.5 and *p* vaule <0.05, a total of 48 differential metabolites have been recognized, whithin l-isoleucine, L-leucine, L-threonine, and dihydroxyacetone were notably enriched in valine, leucine and isoleucine biosynthesis, glycerolipid metabolism, and aminoacyl-tRNA biosynthesis pathways. In brief, trehalose can efficiently improve membrane structural integrity and motion parameters in buck sperm after thawing, and it exerts a cryoprotective impact with the aid of changing sperm amino acid synthesis and the glycerol metabolism pathway.

## Introduction

Semen cryopreservation technology can prolong the survival time of sperm and facilitate long-term preservation and transportation of sperm, and it is of great significance to the promotion of artificial insemination, the acceleration of breeding and the protection of genetic resources. However, the cryopreservation procedure for buck semen results in many types of freezing damage, and decreased sperm viability and survival rates after thawing (Watson, 2000; Sung-Jae et al., 2015, 2016). In addition, the freezing and thawing process can lead to metabolic damage to the sperm so that the frozen and thawed sperm cannot produce enough energy to support the movement of the sperm to the tubal ampullary (Kaabi et al., 2006), greatly reducing the conception rate, which restricts the use of frozen buck semen for artificial insemination.

Trehalose is a naturally occurring disaccharide that is often used as a nonpermeable cryoprotectant for the cryopreservation of semen of many species, such buffalos (Iqbal et al., 2016), rams (Bittencourt et al., 2018; Bucak et al., 2021), bull (Öztürk et al., 2017), and boars (Rukmali et al., 2015; Pezo et al., 2020). However, the mechanism of trehalose in cryopreservation of mammalian sperm remains unclear. Regarding the cryoprotective effect of trehalose on sperm, studies have shown that trehalose cannot enter sperm cells and will form a hyperosmotic environment outside the cell, reduce the formation of intracellular ice crystals, and increase the fluidity of the sperm plasma membrane by reducing the membrane transition temperature. Stabilized biological macromolecules, including proteins, lipids and DNA, effectively protect sperm from cold damage (Aksan and Toner, 2004; Tuncer et al., 2010; Iqbal et al., 2017; Bucak et al., 2020; Olgenblum et al., 2020). It has been concluded that the antioxidant properties of trehalose may be related to its effectiveness in membrane cryopreservation (Lavara et al., 2017). However, a strong functional correlation between 6-phosphogluconate dehydrogenase and fructose-bisphosphate aldolase A isoform 1 was observed in ram sperm in the presence of trehalose, possibly because the process of sperm glycolysis is crucial (Jia et al., 2021). We hypothesized that the cryoprotective effect of trehalose on buck sperm may be related to changes in sperm metabolism. The metabolites of sperm characterize the metabolic regulation of the sperm functional genome, transcriptome, and proteome during the frozen-thawing process (Long, 2020). Therefore, metabolomics technology was used to study small-molecule compounds of sperm metabolites, and more closely approximating the actual phenotype affected by multiple physiological changes has great potential for identifying biomarkers of male fertility (Deepinder et al., 2007; Velho et al., 2018; Memili et al., 2020).

To our knowledge, the present study was to analyse the metabolic characteristics of buck sperm freezing and thawing medium for the first time by using GC-MS-based non-targeted metabolomics technology, and to reveal the metabolic pathway of sperm cryopreserved with trehalose, which will allow us to further understand the cryoprotective effects of trehalose.

## Materials and methods

### Ethics statement

The environment of the cashmere goat farm met the relevant requirements of the experimental facilities in the Chinese national standard “Experimental Animal Environment and Facilities” (GB14925-2010). Health status, pathogenic microorganism infections, and zoonotic infections were monitored to ensure animal safety. All animals were raised under the same management conditions and received the same nutrition. All experimental procedures were conducted per the guidelines of the Inner Mongolia Agricultural University Animal Ethics Committee.

### Semen collection

Semen samples from 20 Inner Mongolia cashmere goats were provided by Inner Mongolia JinLai Animal Husbandry Technology Co., Ltd. (Hohhot, Inner Mongolia). Semen was collected with an artificial vagina and evaluated for rapid wave motion, motility (>75% motility) and concentration (>1.0 × 109 spermatozoon/ml) under a phase-contrast microscope (Olympus BX51).

### Cryopreservation approaches

**FIGURE 1:**
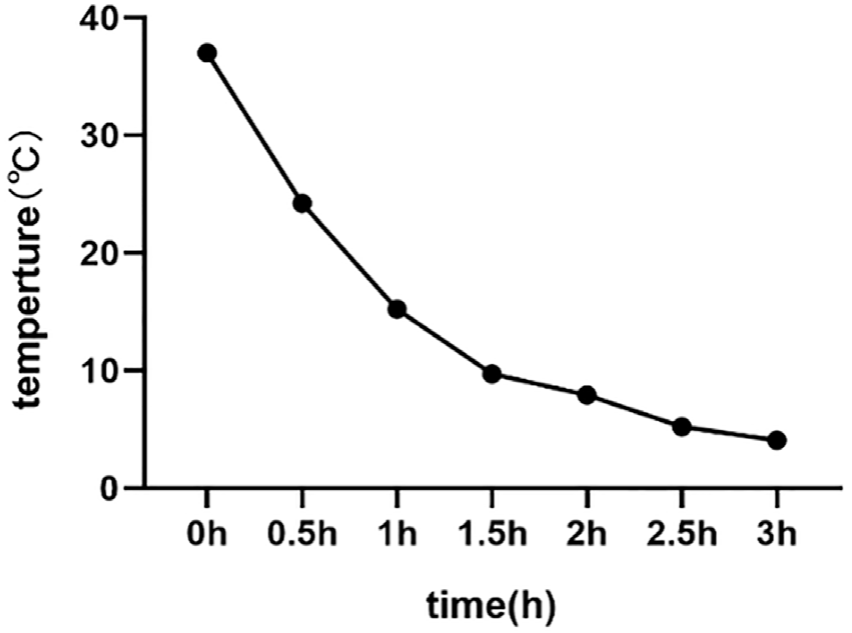
The cooling curve during buck semen cryopreservation.

All chemicals except antibiotics were purchased from Sigma–Aldrich. The basic freezing extenders consisted of 300 mM Tris, 95 mM citric acid, 56 mM glucose, 10% (v/v) egg yolk, 1% glycerol, and 1% antibiotics (v/v, Gibco). The ejaculates were split into two equal aliquots: one aliquot was diluted with basic freezing extenders to a final concentration of 20×10^6^ (the G group), and another aliquot was diluted with basic freezing extenders to 50 mM trehalose (the T group). The extended semen samples were then chilled to 5°C over 3 h; the cooling curve is shown in Figure 1. Then, the sperm suspension was frozen in straws, after 200 µl samples were loaded into prechilled 0.25 ml straws (IMV, France) and sealed with polyvinyl chloride powder. The straws were placed equidistantly on a steel frame and frozen in liquid nitrogen vapour at 4 cm above the surface of liquid nitrogen for 7 min and then submerged in liquid nitrogen and stored until assessment. After storage for 1 week, the straws were thawed in a 38°C water bath for 30 s with agitation. A small fraction of the frozen-thawed semen was used for the assessment of quality. The thawed semen was centrifuged at 1,250 r/min at 4°C for 5 min, and the supernatant was stored at −80°C as a sample for later metabolomic analysis.

### Evaluation of sperm motion parameter

Sperm motility evaluation was performed using a computer-assisted semen analyser (CASA, IVOS II, IMV, France) at 0.25 × 10 magnification. Three microlitres of frozen-thawed semen sample were prewarmed to 37°C in a Leja slide analysis chamber and immediately transferred to the CASA. The sperm total motility (TM; %) and progressive motility (PM; %) were determined by assessing five microscopic fields in at least 1,000 sperm cells.

### Measurement of sperm cell characteristics using flow cytometry

Sperm cell characteristics were investigated by flow cytometry (NovoCyteTM, ACEA, China), which was equipped with a 488 nm blue all-solid-state laser, a 640 nm red all-solid-state laser, and a photomultiplier tube to ensure the stability of the results. The four fluorescence channels, fluorescein isothiocyanate (FITC), PE, PerCP, and APC, could be monitored simultaneously. Acquisitions were performed using NovoExpress software (ACEA Biosciences, China). The detection parameters included the area of all channels (A), width (W), height (H), and time, which could effectively distinguish adhesion cells, cell debris and single cells. A total of 20,000 events were analysed for each semen sample.

Sperm plasma membrane integrity (PMI) was assessed by an Annexin V/PI apoptosis detection kit I (Becton, Dickinson and Company, United States) using the protocol described by Bunel et al. (2014). A portion of 1× binding buffer was used to dilute frozen-thawed semen to 1.2 × 10^6^ cells/ml, which was then stained with 5 µl of Annexin V-FITC and incubated in the dark for 10 min. The sperm cells were subsequently stained with 5 µl of PI, incubated for 5 min in the dark, and suspended in 1× binding buffer prior to flow cytometric detection. The excitation wavelength was 488 nm, and the emission wavelengths were 530 and 630 nm.

Sperm acrosome integrity (AI) was assessed by FITC peanut (*Arachis hypogaea*) agglutinin (Genmed Scientifics, Inc. United States). Frozen-thawed semen was diluted to 1.2 × 107, stained with 150 μl of PNA-FITC staining solution, and incubated for 15 min in the dark. Sperm cells were subsequently stained with 200 µl of PI (0.4 μg/ml) staining solution, incubated in the dark for 5 min, and suspended in phosphate-buffered saline (PBS) (Mocé et al., 2010). Flow cytometry was performed to evaluate sperm AI.

DNA structural integrity (DSI) was assessed with an acridine orange (AO) detection kit. OA buffer (1×) was used to dilute frozen-thawed semen to 1.2 × 107, and the diluted semen was then stained with 10 µl of AO staining solution and incubated at room temperature for 15 min in the dark (Claassens et al., 1992). Flow cytometry was performed to assess sperm DSI after suspension of the stained sperm in PBS. The excitation wavelength was 488 nm, and the emission wavelengths were 530 and 630 nm.

### Metabolite extraction

All the freeze-thawed media samples from T and G group were thawed at 4°C, 80 μl of each sample were removed and transferred to 1.5 ml centrifuge tube, 10 μl of 2-chloro-l-phenylalanine at a concentration of 0.3 mg/ml was added as the internal standard, 600 μl of methanol: acetonitrile (2:1 v/v) was mixed with each sample, and ultrasonicated extraction at −20°C ice water bath for 5 min, the tubes were centrifuged at 12,000 rpm for 10 min at 4°C to collect the supernatants, following by combining aliquots of all samples to prepare Quality control (QC) samples. The supernatant from each sample was vacuum dried at room temperature. 80 μl of methoxyamine hydrochloride (soluble in pyridine, 15 mg/ml) was added to the sample. After vortexing for 2 min, the mixtures were incubated at 37°C for 90 min, and then added 80 μl of BSTFA (containing 1% TMCS) and 20 μl of n-hexane for 2 min and react at 70°C for 60 min. All samples were stored at ambient temperature for 30 min previous for GC-MS analysis.

### Gas chromatography-mass spectrometric analysis

Metabolic analysis of the samples and internal standard was performed on an Agilent 7890B gas chromatography system coupled to an Agilent 5977A MSD system (Agilent Technologies Inc., CA, United States). Chromatographic separation was performed on a DB-5MS fused-silica capillary column (30 m × 0.25 mm×0.25 μm, Agilent J&W Scientific, Folsom, CA, United States). Helium carrier gas was used at a constant flow rate of 1 ml/min through the column.1 μl of derivatized mixture was injected in splitless mode, the injector temperature was maintained at 260°C. The oven was programmed initially at 60°C, ramped to 125°C at a rate of 8°C/min, to 210°C at a rate of 5°C/min, to 270°C at a rate of 10°C/min, to 305°C at a rate of 20°C/min, and finally held at 305°C for 5 min. The collision energy was 70 eV. Mass spectrometric data was acquired in a full-scan mode (m/z 50–500).

### Data analyses processing

Analysis Base File Converter software was utilized to transform the raw data (D layout to Abf format), and then the abf datas have been imported into MD-DIAL software for data processing. Metabolites were identified through the LUG database (untargeted metabolite database for GC–MS from Lumingbio). The parameters of the models, which includes R2X, R2Y, Q2Y, and the permutation test effects have been calculated to assess the satisfactory of the multivariate models and keep away from the risk of overfitting. Data had been transformed with the aid of log10, and the ensuing information matrix have been then imported into R ropls package. Principle component analysis (PCA) an (orthogonal) partial least-squares-discriminant analysis (O) PLS-DA were accomplished to visualise the metabolic difference amongst experimental groups, after suggest centering and unit variance scaling. Variable importance in projection (VIP) values obtained from the OPLS-DA model and *p* values from a two-tailed Student’s *t* test was calculated to identify the potential metabolites, and those with VIP values >1.5 and *p* values <0.05 were considered differential metabolites. Identification of potential metabolic pathways had been performed using the pathway analysis module MetaboAnalyst 5.0 for metabolomic studies and utilizing Kyoto Encyclopaedia of Genes and Genomes (KEGG) database as a knowledge base.

### Statistical analysis

All data of the sperm motion parameter and flow cytometry variables were estimated and are shown as the means ± standard deviation (SD). ANOVA was conducted in SAS 9.4 (SAS Institute Inc., Cary, NC, United States) to determine the significance of differences between the T and G groups with *p* < 0.05.

## Results

### Effects of trehalose on the physiological characteristics of buck sperm after cryopreservation

The effects of trehalose on frozen-thawed sperm total motility, progressive motility, acrosome integrity, plasma membrane integrity and DNA structural integrity were evaluated (Table 1). Trehalose (50 mM) was associated with higher values of sperm quality after thawing (*p* < 0.01).

**TABLE 1:** Effects of 50 mM trehalose addition on sperm characteristics after freeze—thawing. The data are presented as the mean ± SEM; values with different superscripts within a row differ significantly (*p* < 0.01). TM, total motility; PM, progressive motility; PMI, plasma membrane integrity; AI, acrosome integrity; DSI, DNA structural integrity.

	G (control)	T (50 mM trehalose)
TM (%)	36.8 ± 1.54^a^	68.7 ± 0.86^b^
PM (%)	20.3 ± 1.42^a^	45.7 ± 1.37^b^
PMI (%)	47.43 ± 1.13^a^	72.03 ± 1.5^b^
AI (%)	58.75 ± 2.04^a^	86.72 ± 1.66^b^
DSI (%)	80.13 ± 2.28^a^	96.97 ± 0.57^b^

Values with different ^a^ and ^b^ within the same row indicate significant differences.

### Multivariate statistical analysis of metabolic profiles

**FIGURE 2:**
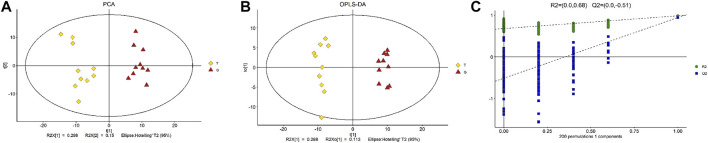
Multivariate statistical score graph. **(A)** PCA score plot of differential metabolites. **(B)** OPLS-DA score plot of differential metabolites. **(C)** Statistical validation with permutation analysis (200 times) of the corresponding PLS-DA model. R2 is the explained variance, and Q2 is the predictive ability of the model.

We determined obvious metabolomic differential metabolic characteristics of the thawing media by utilizing the GC–MS metabolomics analysis, between the groups treated with 50 mM trehalose supplementation and without trehalose. Unsupervised PCA had been used to observe the overall distribution of the samples and the stability of the overall analytical process. The PCA score plots display distribution trendencies between the T and G group, with all of the observations falling inside the 95% Hoteling T2 ellipse (R2X = 0.551). PCA was initially performed on the entire data set to explore clustering in the samples. There appeared to be a clear segregation of the metabolomic profiles of the thawing media between the T and G groups, and the trend of intergroup separation was significant (Figure 2A). Subsequently, a supervised OPLS-DA model was used to determine the difference between the thawing media metabolome of the T group and the G group (Figure 2B). The performance traits of this multivariate version from a descriptive and predictive factor of view were R2X = 0.401, R2Y = 0.984, and Q2 = 0.934. The OPLS-DA model was also validated and passed the permutation test, which showed no observable overfitting (Figure 2C). The score plot shows a distinct segregation of the two groups of samples, indicating that the metabolic differences were significant.

### Candidate metabolite biomarker screening and pathway analysis

The VIP scores obtained by utilizing OPLS-DA analysis can be used to measure the impact and explanatory capacity of metabolite expression patterns on the classification and discrimination of each group of samples. A total of 48 metabolites with VIP scores greater than 1.5 and *p* < 0.05 were identified as differential metabolites. Among them, there were 24 differential metabolites with KEGG IDs and HMDB IDs (Table 2).

**TABLE 2:** The differential metabolites in the freezing media for the T and G groups.

Metabolite name	Kegg ID	Total similarity	HMDB	Sub class	VIP	*p*-value
Glycolic acid	C03547	991.2844	HMDB0000115	Alpha hydroxy acids and derivatives	1.82203045540948	1.00377257617081E-13
Allantoic acid	C00499	789.3102	HMDB0001209	Amino acids, peptides, and analogues	1.81426158212667	6.94178289715613E-12
L-leucine	C00123	764.5416	HMDB0000687	Amino acids, peptides, and analogues	1.81364472212532	2.26282940343527E-10
N-acetylornithine	C00437	870.6558	HMDB0003357	Amino acids, peptides, and analogues	1.81166495220627	1.36697081773895E-11
N-acetylputrescine	C02714	892.2279	HMDB0002064	Carboximidic acids	1.80799808987634	7.28793569065889E-12
3-hydroxypropionic acid	C01013	965.0267	HMDB0000700	Beta hydroxy acids and derivatives	1.79524073613078	1.53462160012709E-11
Glyceric acid	C00258	907.4557	HMDB0000139	Carbohydrates and carbohydrate conjugates	1.78987068604703	9.87003171156282E-12
Cholesterol	C00187	811.4595	HMDB0000067	Cholestane steroids	1.73748567656342	3.08313549279416E-09
Dihydrocholesterol	C12978	740.6195	HMDB0001569	Cholestane steroids	1.72392097143653	2.95441940524306E-08
Inulotriose	C01355	851.2805	HMDB0003539	Carbohydrates and carbohydrate conjugates	1.69299589144546	3.98503392994622E-08
Delta-tocopherol	C14151	475.3806	HMDB0002902	Quinone and hydroquinone lipids	1.68664323887317	1.00149600884067E-07
5-aminovaleric acid	C00431	843.8047	HMDB0003355	Amino acids, peptides, and analogues	1.6493244909004	2.12175572834716E-07
Chlorogenic acid	C00852	803.7262	HMDB0003164	Alcohols and polyols	1.64296587714703	6.23724832090101E-07
5-methoxytryptamine	C05659	918.1883	HMDB0004095	Tryptamines and derivatives	1.63842189770607	9.30753873330454E-07
L-threonine	C00188	993.6814	HMDB0000167	Amino acids, peptides, and analogues	1.61776921989435	1.94910862115471E-06
Dihydroxyacetone	C00184	852.5062	HMDB0001882	Carbohydrates and carbohydrate conjugates	1.61327254531687	3.03434938467899E-06
Gamma-aminobutyric acid	C00334	858.1658	HMDB0000112	Amino acids, peptides, and analogues	1.60723968680844	2.7483304629845E-07
Deoxycholic acid	C04483	773.2537	HMDB0000626	Bile acids, alcohols and derivatives	1.58467513763386	1.69457598383148E-05
L-isoleucine	C00407	959.4128	HMDB0000172	Amino acids, peptides, and analogues	1.57893725345317	8.92161530831423E-06
N-methylalanine	C02721	889.7502	HMDB0094692	Amino acids, peptides, and analogues	1.5681144443649	1.1747551517369E-06
Malonic acid	C04025	780.5543	HMDB0000691	Dicarboxylic acids and derivatives	1.55774115850513	2.15611990388497E-05
4a-carbinolamine tetrahydrobiopterin	C00268	758.2062	HMDB0002215	Pterins and derivatives	1.54931830032016	1.91783364960654E-05
Galactinol	C01235	876.1626	HMDB0005826	Carbohydrates and carbohydrate conjugates	1.53607940973879	6.6491357243501E-05
Galactose	C00984	986.1047	HMDB0000143	Carbohydrates and carbohydrate conjugates	1.5156310023485	2.32991554190241E-05

**FIGURE 3:**
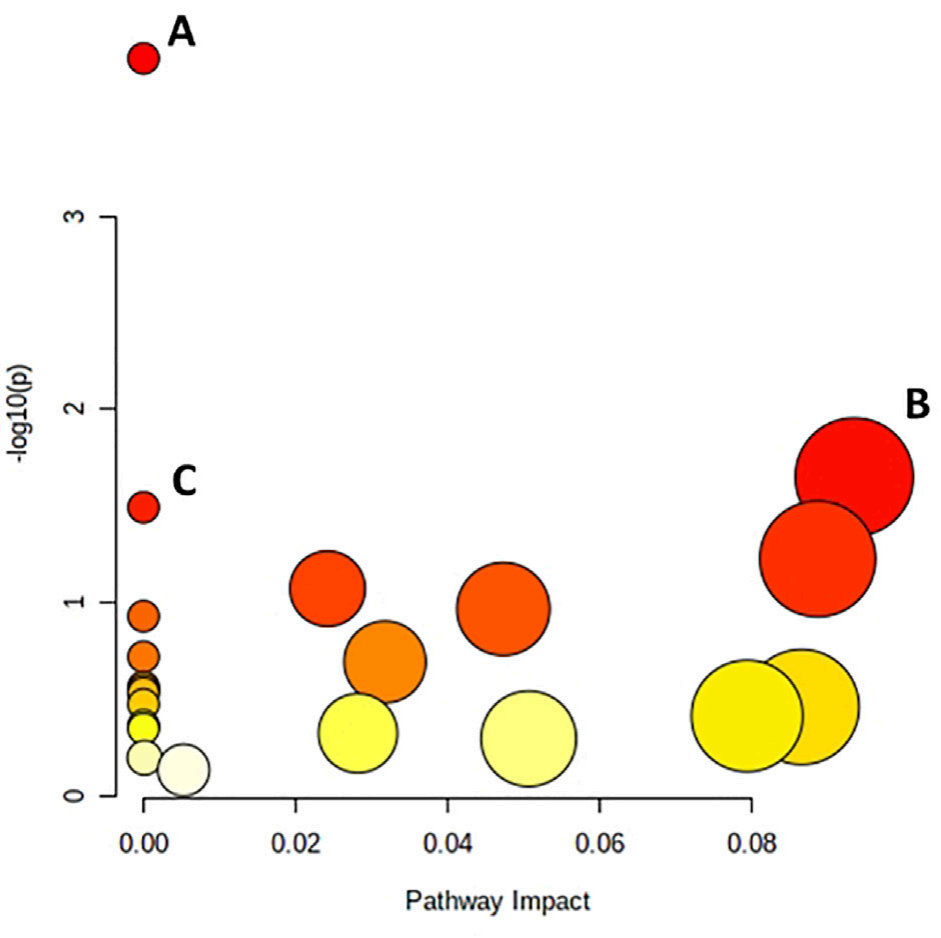
Pathway analysis of metabolites present in the thawing media of the T and G groups. **(A)** Valine, leucine and isoleucine biosynthesis; **(B)** Glycerolipid metabolism; **(C)** Aminoacyl-Trna biosynthesis.

The differential metabolites were evaluated to identify the pathways to which they belong (Figure 3). The pathway impact was derived from pathway topological analysis, which suggests the importance of the output pathway for general metabolism. The potentially affected pathways were the valine, leucine and isoleucine biosynthesis, glycerolipid metabolism, and aminoacyl-tRNA biosynthesis metabolism pathways, in which differential metabolites were significantly enriched.

## Discussion

**FIGURE 4:**
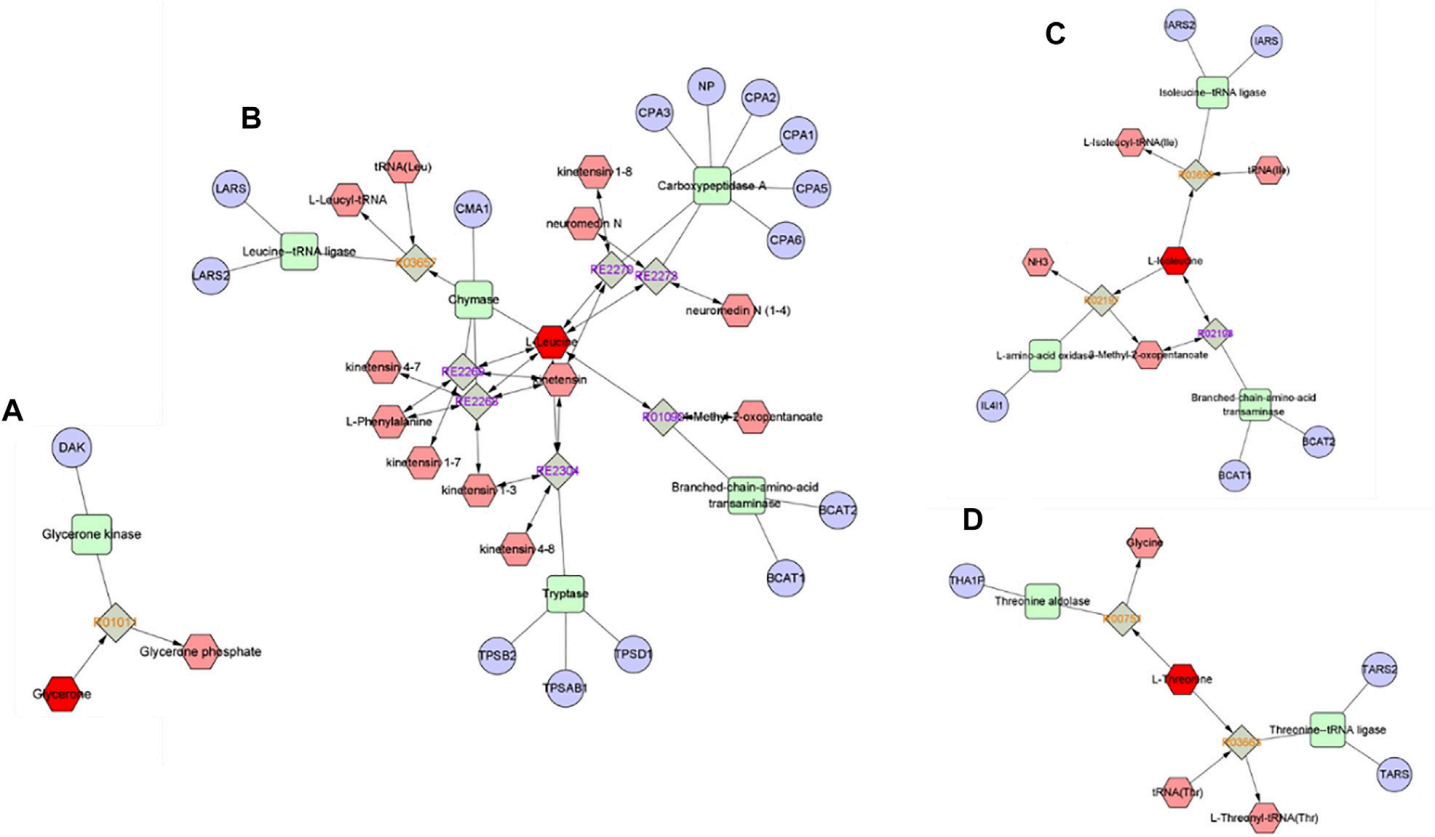
Pathway and network analyses of the metabolites were performed using Metascape. **(A)** Dihydroxyacetone/glycerone **(B)** Leucine **(C)** Isoleucine **(D)** Threonine. The metabolites are shown in red hexagons. Gray square: Reaction node with reaction ID; Pale red hexagon: Compound node; Green square: Enzyme node; Blue circle: Gene node.

**FIGURE 5:**
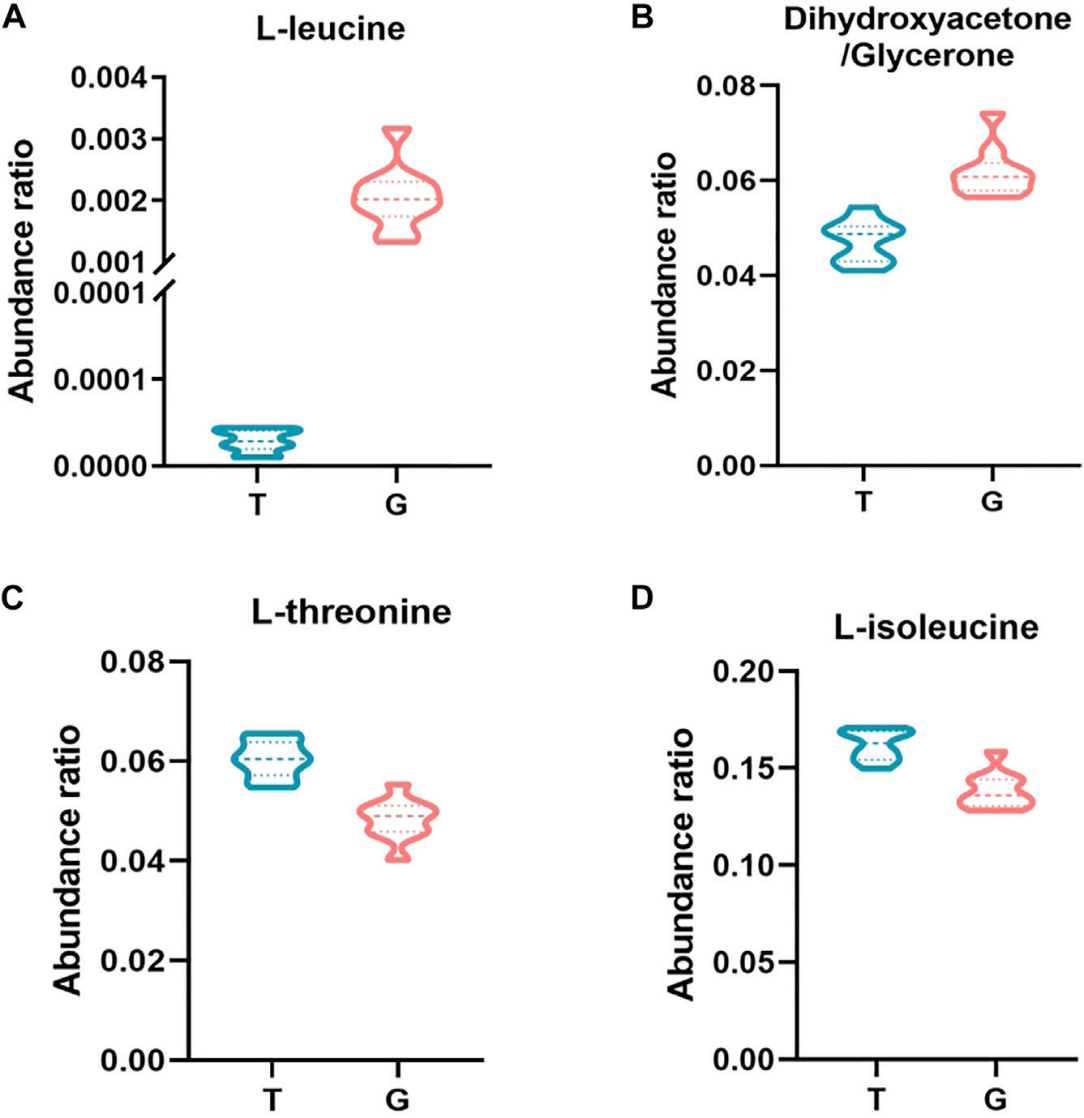
Abundance ratio of important metabolites in the freezing media of the T and G groups. **(A)** Dihydroxyacetone/glycerone. **(B)** Leucine. **(C)** Isoleucine. **(D)** Threonine. The difference was significant at the 0.01 level between the T and G groups.

The cryoprotective effect of trehalose has been elucidated in studies of sperm from many mammalian species, such as boars (Gómez-Fernández et al., 2012), bulls (Tuncer et al., 2011), rabbits (Zhu et al., 2017), and rams (Bucak and Tekin, 2007; Bucak et al., 2020; Bucak et al., 2022). Although the exact mechanism of action of trehalose is not fully understood, but it is suggested that trehalose protects cells by increasing the surface tension of the extender and stabilizing the plasma membrane, which is due to the direct interaction of trehalose with the polar headgroups of cell membrane phospholipids (Iqbal et al., 2018), and decrease reactice oxygen species generated and reduce lipid peroxidation during cryopreservation through its own antioxidant effects (Zhu et al., 2017). Glycerol is usually used as a permeable protectant for sperm freezing (Başpınar et al., 2011; Bucak et al., 2013), but it can have an adverse toxic effect on the metabolism and plasma membrane of frozen thawed sperm, so the glycerol concentration is reduced by adding high molecular weight cryoprotectants and antioxidants to the freezing extender (Swelum et al., 2011; Öztürk et al., 2020). In this study, the trehalose had a beneficial synergistic effect with low-concentration glycerol, which can significantly increase buck sperm total motility and progressive motility after thawing and preserve sperm structural integrity, which was consistent with the findings of previous studies (Bucak et al., 2007; Akhtarshenas et al., 2018; Öztürk et al., 2020). However, optimum cryopreservation requires maintaining metabolic function, and metabolomic approaches allow the study of cellular metabolites, which are thought to be closer to actual phenotypes than proteomes or genomes (Sousa et al., 2020). Therefore, this study used GC–MS-based untargeted metabolomic analysis technology to observe the effect of trehalose on the metabolome of ram frozen semen, which will help us understand the mechanism of the cryoprotective effect of trehalose on sperm (Panner Selvam et al., 2019). The results showed that trehalose affects buck sperm metabolism during cryopreservation, and the PLS-DA score plot of the freeze–thawed medium for the trehalose-added group and control group exhibited a clear difference. Pathway enrichment and topological analysis of metabolic pathways revealed that valine, leucine and isoleucine biosynthesis, glycerolipid metabolism, and aminoacyl-tRNA biosynthesis were closely related to the cryoprotective effect of trehalose, and the significantly enriched metabolic pathway hit 4 differential metabolites, including L-isoleucine, L-leucine, L-threonine, and dihydroxyacetone (Figures 4, 5).

Isoleucine and leucine, as branched-chain amino acids, can activate cells and play an important role in various physiological and metabolic functions, such as protein metabolism, lipid metabolism, glucose metabolism and transportation (Gu et al., 2019). In this study, the abundance ratio of L-isoleucine in the trehalose-treated group was higher than that in the thawing media from the G group. In the report of Mateo-Otero et al., isoleucine was inversely correlated with progressive motility of pig spermatozoa during liquid storage (Mateo-Otero et al., 2021). Significant enrichment of unique proteins in poor quality spermatozoa was also observed in buffalo to isoleucine and leucine degradation pathways (Binsila et al., 2020). There was a positive correlation between membrane integrity and the concentrations of isoleucine (Santiago-Moreno et al., 2019). This suggests that isoleucine may also have a positive impact on sperm motility and structural integrity after thawing, but further research was need on isoleucine supplementation on sperm performance. The L-leucine abundance ratio was higher in the thawing media of the control. A study by Zhang et al. (2017) showed that leucine had a positive effect on the motility of zebrafish sperm. Significantly lower concentrations of leucine have also been shown in low fertility bull spermatozoa (Velho et al., 2018). In a study by Kai et al. (2018), asthenozoospermic patients (men with poor sperm motility) had reduced levels of leucine. Leucine may be beneficial to sperm motility after thawing, however the excessive abundance of leucine within the thawing media of the control can be due to severe damage to the sperm membrane structure, ensuing inside the efflux of intracellular material. Therefore, trehalose may maintain post-thawed sperm motility and membrane integrity by affecting the anabolism of isoleucine and leucine in buck sperm thoughtout cryopreservation.

The abundance ratio of threonine in the trehalose-treated group was higher than that in the thawing media from the G group. Threonine is an essential amino acid in mammalian cells. Apart from its role in protein synthesis, threonine can be catabolized to generate metabolic building blocks for a wide range of biosynthetic reactions, such as glycine, acetyl-CoA, and pyruvate (Chen and Wang, 2014). As the principal compound in accessory sex gland fluid, threonine metabolism might be crucial for male reproductive physiology (Thurston et al., 1982; Fröhlich et al., 2010), According to the research, dietary supplementation with threonine can improve boar spermatozoa quality and fertility rates after insemination (Dong et al., 2016). This indicates that threonine may play a positive role during buck semen cryopreservation, and its protectant mechanisms may be related to amino acids involve in binding phospholipid membrane bilayers and stabilizing the cell membranes (Anchordoguy et al., 1988; Bilodeau et al., 2001).

The capability of sperm from many species to utilize glycerol had been well established (Mann and White, 1957; Mohri et al., 1970). As an intermediate product of glycerol metabolism, Dihydroxyacetone was an intermediate product of glycerol metabolism, and readily incorporated into sperm lipids for utilization (Selivonchick et al., 1980). This may be the reason for the lower abundance ratio of dihydroxyacetone in the freezing medium of the trehalose-supplemented group in this study. When dihydroxyacetone was the substrate, dihydroxyacetone phosphate and fructose-1,6-bisphosphate accumulated (Jones et al., 1992), and involved in glycolysis for producing and providing ATP for spermatozoa motion (Nakamura et al., 1982; Ford, 2006; Fu et al., 2019). Therefore, the cryoprotective effect of trehalose may alter glycerol metabolism during cryopreservation of buck sperm, In conclusion, this study evaluated the effect of trehalose on the metabolism of buck sperm during cryopreservation, and identified 48 differential metabolites. According to bioinformatics analysis, trehalose may affect the amino acid synthesis and glycerol metabolism of buck sperm, and its cryoprotection maintains post-thawed buck sperm motility and structural integrity. In addition, some metabolites, such as L-isoleucine, L-leucine, L-threonine, and dihydroxyacetone may serve as potential biomarkers for assessing the quality of buck sperm after thawing. (Sousa et al., 2020).

## Data Availability

The datasets presented in this study can be found in online repositories. The names of the repository/repositories and accession number(s) can be found below: The data are available from the corresponding author upon request. The raw data for the present study were submitted to the online public repository MetaboLights with the unique identifier MTBLS4318 (www.ebi.ac.uk/metabolights/MTBLS4318).
